# Measuring patients' satisfaction toward health tourism in Malaysia through hospital environment, nutritional advice, and perceived value: A study on Chinese exchange students

**DOI:** 10.3389/fpubh.2022.893439

**Published:** 2022-08-09

**Authors:** Liu Li, Neethiahnanthan Ari Ragavan, Ataul Karim Patwary, Wu Baijun

**Affiliations:** ^1^School of Management, North Minzu University, Yinchuan, China; ^2^Faculty of Social Sciences and Leisure Management, Taylor's University, Subang Jaya, Malaysia; ^3^Faculty of Hospitality, Tourism and Wellness, Universiti Malaysia Kelantan, Kota Bharu, Malaysia; ^4^School of Marxism Studies, Chengde Medical University, Chengde, China

**Keywords:** patients' satisfaction, health tourism, hospital environment, nutritional advice, perceived value

## Abstract

Malaysia is a reputed destination for health tourism in the Asia Pacific region for its hospitable nature and good quality of service. Patients' satisfaction has been a key concern in health tourism to maintain the flow of tourists' arrival for medical purposes. By considering the importance of health tourism and patients' satisfaction in Malaysia, this study aimed to examine the influence of hospital environment, nutritional advice, perceived value on patients' satisfaction. This study is cross-sectional in nature and follows a quantitative approach. The researchers used questionnaires as a survey tool to obtain information from the respondents. The sample of this is chosen from Chinese exchange students in Malaysia. Using a systematic random sampling technique, 205 usable responses were selected from the respondents and proceeded with further analysis. The study conducted structural equation modeling using Smart PLS version 3. The results found that hospital environment, nutritional advice, and perceived value significantly influence patients' satisfaction in Malaysia.

## Introduction

Researchers and marketing experts have focused their attention on the relevance of customer satisfaction to a company's long-term survival and growth. If anyone wants to acquire a competitive edge in today's business world, to pay attention to the quality of their services. Increasingly, customer orientation is being applied to the healthcare business. As health is seen as one of the most important human assets, it's not unusual for people to travel for (1). Health is more than just physical wellbeing. A person's physical, mental, and social wellbeing are all included in the term “health” when seen from a holistic viewpoint instead of only the absence of sickness or illness (2). Tourism is any activity that involves a person visiting or living outside usual surroundings for pleasure, business, or any other reason for less than a year in a row. It might be argued that the term “tourism” encompasses more than only recreational travels, such as commercial and medical trips, as well as travels for pleasure. Travels associated with health services may be either curative (medical) or preventative, or wellness-oriented, depending on the purpose of the trip (3). Traveling from one location to another to get health-related services might be referred to as health tourism under several situations (4). Aside from obtaining health-related treatments, visitors who go on a health-related vacation may also profit from the many tourist attractions they come across. Such considerations have little impact on health tourists' core goal of improving their quality of life via access to medical treatment (5). No single researcher has been able to agree on what exactly constitutes health to tourism. According to Abbas et al. (6), the term encompasses a wide range of activities, including medical care to health assessments to surgery to beauty treatments to rehabilitation to convalescence to leisure and recreation at a particular location. In addition, medical tourism, medical wellness tourism, and wellness tourism are all separate subcategories within the author's overall classification of health tourism. Health tourism encompasses both medical and wellness travel (7). According to Pan et al. (8), an accurate definition of health tourism includes medical and wellness tourism. Health tourism is a fast-growing segment of the tourist industry (9). Low-cost remedial methods, more advanced hi-tech services and products globally trained competent medics and nurses, and amazing healthcare facilities are all factors that encourage health tourists to seek treatment outside their nation. As a result, many countries' tourist businesses, particularly developing countries, see health tourism and associated fields as one of the most profitable segments (10, 11). Medical travel abroad has become more accessible and less expensive due to globalization in the last several decades. Health care delivery is already beginning to cross national borders, further demonstrating that globalization is no longer limited to manufacturing. Many developing countries are now leading destinations for medical tourism, thanks to globalization (12). According to a literature assessment, developing nations are drawing many health tourists. The biggest reason people seek healthcare in developing countries is cost (13). Asia has recently risen to the top of the most popular locations for medical tourists (14).

**Figure 1:**
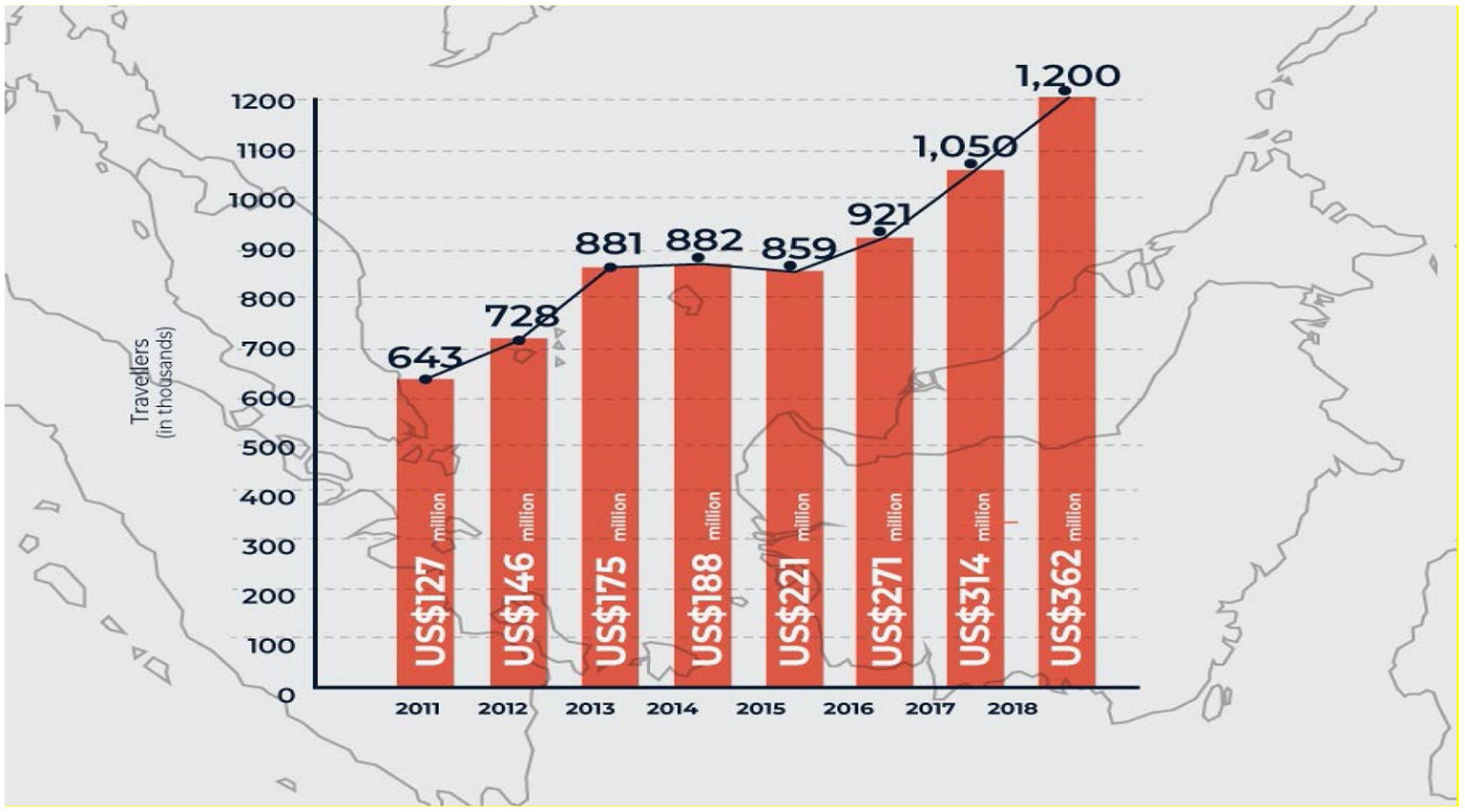
Health Tourism Market in Malaysia. Source: The Malaysia Healthcare Travel Council (MHTC), 2019.

Figure 1 shows the uprise of Malaysia's market since 2018. It can be seen that it has been continuously rising from 2011 till 2018.

Nutritional advice is based on dietary needs established by published research and clinical guidelines (15). The goal of hospital environments refers to the spatial, physical, and functional design characteristics that healthcare environments should possess to achieve two goals, including lowering the stress level, which can be quite significant for both staff and patients. Malaysia is a key player in health tourism (16). Healthcare tourism brought in RM 588.6 million in 2015 and attracted 859,000 visitors to Malaysia (17). The government's goal of making Malaysia a regional center for medical tourism has made health tourism a top priority (18). However, Malaysia is one of the world's most popular tourist destinations (19). It has yet to establish itself as the region's top destination for health tourism. The government is making significant efforts to boost the country's health tourism business. For Malaysia's healthcare business, identifying the underlying elements that may influence patients' satisfaction with health tourism has arisen as a critical concern. There hasn't been a thorough investigation on its suitability as a health tourist attraction yet. Patients' satisfaction with health tourism in Malaysia has received a cursory look at the aspects that may influence their experience. Health tourism is a multifaceted industry that requires careful consideration of factors such as cost, service quality, motive, destination image, and perceived value (20). Kurgun et al. (21) have recommended that hospital environment, perceived value is important element in tourism industry which ultimately led to satisfaction level. Medical advice is one of the most important aspects contributing to customer satisfaction with health tourism (22). To get a whole picture of how patients feel about health tourism, it's necessary to consider these factors. According to a study of the literature, it has been found that few studies have assessed the simultaneous influence of these variables on patients' satisfaction with health tourism. Consequently, by considering the importance of health tourism and patients' satisfaction in Malaysia, the current research was designed to examine the influence of hospital environment, nutritional advice, perceived value on patients' satisfaction.

## Literature review

### Hospital environment and patients' satisfaction

There are two types of quality in health care: technical (or result) and functional (or process) (23). Functional quality focuses on how the service is delivered to customers rather than what they get. As a result, a patient's quality of care is measured by the correctness of their diagnosis and treatments and how the care is given to them (24). But Dewa et al. (25) argue that quality is not described in terms of clinical quality but rather service delivery quality in health care. It is important for a hospital to not simply concentrate on clinical outcomes. It can count on the work being of the highest quality and would go to a hospital if they can't rely on the doctors to provide them with top-notch care. Instead, the focus should be on giving high-quality service, aside from special medical treatment. The focus should be paid to establishing proper interaction between staff and patients, or in general, connecting with patients (26). From the patient's standpoint, service quality involves the impression of medical treatment and physical facilities and relationships with both medical and nursing staff members.

Based on above literature, this following hypothesis can be posed as:

*H1: Hospital environment is positively related with Patients' satisfaction*

### Nutritional advice with patients' satisfaction

People's post-consumption evaluation of a product or service may be characterized as satisfaction (27). In tourism, visitors' emotional states are often linked to their feelings of pleasure, and it is widely assumed that visiting the place of their choosing would result in a feeling of contentment (28). As a result, customer satisfaction is comparable to the joy travelers have after visiting a certain location. When visitors' expectations for service are compared to the service they get, contentment results from this comparison (29). Tourists are considered happy if the service they get is in line with what they had hoped. The link between patients' expectations of healthcare providers' services and the service given by healthcare organizations may be regarded as a measure of patient satisfaction. In this case, patients are deemed pleased when the healthcare treatment they get meets or exceeds their expected outcomes. According to an existing research study, several characteristics of health tourism satisfaction may be attributed to it. One of the crucial factors in health tourism is the cost of treatment (30). The degree to which visitors are motivated has also been linked to their level of satisfaction (31, 32). Patients are also satisfied with the destination's image (33). It is for healthcare institutions to meet visitors' needs without delivering high-quality service and value (34, 35). The following hypothesis can be posed as:

*H2: Nutritional advice is positively related with patients' satisfaction*

### Perceived value and patients' satisfaction

To effectively use relationship-based marketing in the tourist industry, a company's perceived worth must be considered (36). Individuals' decision-making is influenced by this, too, to some extent (37). It's all about comparing the product's or service's value to its price that makes up the concept of perceived value (38). In this way, contentment is closely associated with it since it is believed to occur when people think they have achieved a good value. For the most part, perceived value is made up of two factors: first and primarily, the benefits that one receives, which can be in the form of social as well as economic, and secondly, sacrifices, which include the price one must pay for a given product or service and the time it takes one to acquire the service or product (39). Study after study has found that perceived value is a crucial predictor of customer satisfaction. Adly (40) claimed that perceived value is an important factor in customer satisfaction. In addition, the impact of perceived value on visitor satisfaction has been expanded. Numerous studies in tourism have shown that customer satisfaction is closely linked to a destination's perceived value (41, 42). Patients' pleasure was also shown to be influenced by perceived value (43, 44).

The following hypothesis can be posed as:

*H3: Perceived value is positively related with patients' satisfaction*

### Methodology

By considering the importance of health tourism and patients' satisfaction in Malaysia, the current research was designed to examine the influence of hospital environment, nutritional advice, perceived value on patients' satisfaction. This study is cross-sectional and followed a quantitative approach to examine the influence of hospital environment, nutritional advice, perceived value on patients' satisfaction. A cross-sectional study usually involves measuring all variables for all cases within a short period and once only. The researchers used structured questionnaires as a survey tool to obtain information from the respondents. The sample of this is chosen Chinese exchange students in Malaysia. To collect the data, the researchers used a systematic random sampling technique. Two hundred and five usable responses were collected from the respondents and proceeded for further analysis. The initial analysis was conducted using SPSS version 23, and structural equation modeling was conducted using Smart PLS version 3.

### Measurement of the study

One-to-seven-point Likert scales have been used to measure responses (1) Strongly disagree, (2) Disagree, (3) Somewhat disagree (4) No opinion/Neutral (5) Somewhat agree, (6) Agree, (5) Strongly Agree. The measurement of this study adopted from previous studies. For patients' satisfaction was measured using four items from Antreas and Opoulos (45). The items include “I am satisfied with staying in this hospital.” Hospital environment was measured using a three items construct adopted from Markovic et al. (46). The items include “Available and clear information at the hospital.” A five items construct used to measure nutritional advice adopted by Yildiz and Erdogmus (47). The items include “The nutritional advice provided to me is useful.” Perceived value was using six items construct adopted from Sweeney and Soutar (48). The items include “Performing service in the promised time.” These items further tested and validated by Petrick (49).

## Data analyses and hypotheses results

The researchers examined the data using PLS-SEM to assess Hospital Environment, Nutritional Advice, Patients' Satisfaction, and Perceived Value. We report results using a significance level at *p* < 0.01 and *p* < 0.001.

**Figure 2:**
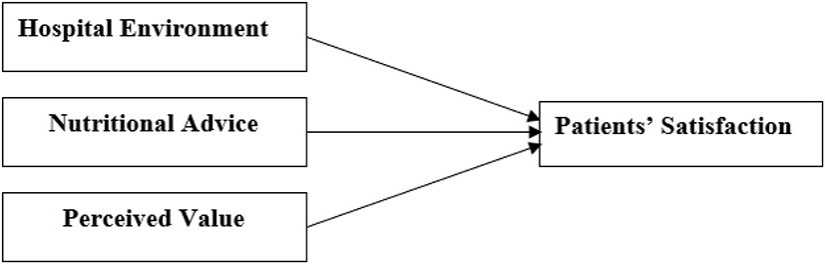
Research framework.

In terms of content validity, a variety of operational aspects were evaluated using pre-existing research and multiple-item measurements. Both confirmatory and exploratory factor analyses confirm the factorability of the variables. An example of this is depicted in Figure 2. One item had a loading above 0.60, and the rest exceeded the recommended 0.70 loading. Table 1 lists all the constructs, and the Cronbach alpha for each is≥0.70.

**Table 1:** Construct validity and reliability.

	**Cronbach's alpha**	**Composite reliability**	**Average variance extracted (AVE)**
Hospital environment	0.865	0.917	0.786
Nutritional advice	0.916	0.937	0.750
Patients' satisfaction	0.871	0.912	0.721
Perceived value	0.881	0.909	0.628

Furthermore, the overall reliability of the composite was above the standard. The average variance extracted was checked to ensure convergent validity and found that every variable was more significant than or equal to the recommended value of 0.50 (see Table 1). As a result, all of the variables point to the validity and dependability of the contents of the questionnaire.

**Figure 3:**
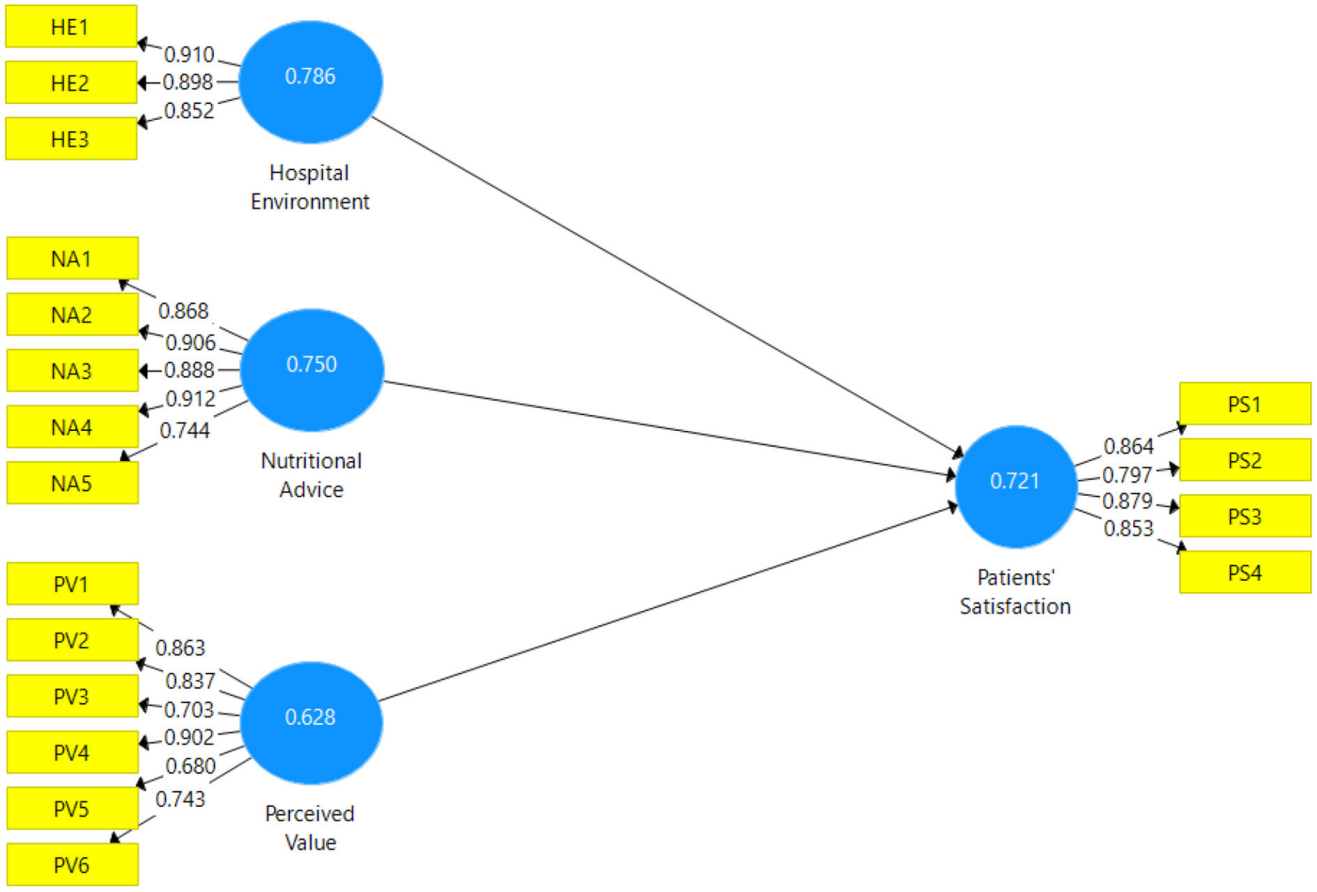
Measurement model.

Figure 3 shows the factor loading of individual items and confirms the confirmatory factor analysis. To evaluate the mdoe, the discriminant validity of the 4-variables is used in the study, Heterotrait-and Monotrait (HTMT) analysis was performed.

The result of confirmatory factor analysis shown in Table 2 supports the empirical evidence of the distinctiveness of the variables. No correlation exceeds the limit of maintaining an HTMT value of 0.85. Thus, all the study variables ensured the discriminant validity for further analysis.

**Table 2:** Discriminant validity (HTMT).

	**Hospital environment**	**Nutritional advice**	**Patients' satisfaction**	**Perceived value**
**Hospital environment**				
Nutritional advice	0.131			
Patients' satisfaction	0.290	0.239		
Perceived value	0.184	0.369	0.229	

### Structural equation modeling

In Smart-PLS, two models are used to examine the effects of independent variables on the dependent variable: the measurement model and the structural model. These include constructing validity and reliability that have already been discussed in detail. R square is also shown in the structural modeling equation for the ability of independent variables to predict the outcomes of their underlying dependent variables. The three variables chosen to have an R-Squared Value of 0.111, indicating a connection between the hospital environment, nutritional advice, and satisfaction. As a further measure of model fit, the SRMR (statistical correlation coefficient) was calculated, which was found to be 0.07.

As demonstrated in Table 3, the hospital environment (β = 0.221, *T* = 3.918, *P* = 0.000), nutritional advice (β = 0.158, *T* = 2.828, *P* = 0.005), and perceived value (β = 0.119, *T* = 2.301, *P* = 0.022) all have a significant influence on patient satisfaction. Hospital environment features have the greatest impact on patient satisfaction. Nutritional advice has a significant positive influence on patient satisfaction. Nutritional advice has a positive effect on patient satisfaction, as does perceived value have a positive effect on patient satisfaction. As a result, all hypotheses are supported.

**Table 3:** The Direct effects of HRM practices on operational performance.

**Hypothesis**	**Original sample (O)**	**Sample mean (M)**	**Standard deviation (STDEV)**	**T statistics (|O/STDEV|)**	**P values**
H1: Hospital environment -> patients' satisfaction	0.221	0.225	0.056	3.918	0.000
H2: Nutritional advice -> patients' satisfaction	0.158	0.160	0.056	2.828	0.005
H3: Perceived value -> patients' satisfaction	0.119	0.126	0.052	2.301	0.022

### Discussion and conclusion

The results found that the hospital environment, nutritional advice, and perceived value significantly influence patients' satisfaction in Malaysia. The results suggest that the responsible stakeholders maintain health tourism in Malaysia by good health care service, hospital environment, appropriate nutritional advice from the doctors, and perceived value of the medical service which is aligned with previous study of Johari and Ong (50). It further concludes that, in terms of service quality, majority of the patients are satisfied with the current services offered by the Malaysian hospitals.

Lam (51) and Manaf (52) found that, it is crucial to keep and attract more medical tourists by providing high-quality service, such as delivering accurate information to patients, up-to-date information services, and patients' awareness of the worth of the money they spent on their treatments. In order to attract and retain medical tourists, the Malaysian government needs to identify the many needs that they can aggressively promote them as a key medical tourism player in order to satisfy their customers' needs (53, 54).

Furthermore, it is necessary to publicize medical success stories and the services they provide in order to attract more overseas patients (55, 56). The findings of this study make it abundantly evident that the competitiveness of a destination is a significant consideration for medical tourists. General tourists and medical tourists are not motivated by the same things (57, 58). Tourists go on vacation to have fun, relax, or take in the sights, and this is known as general tourism.

### Contributions of the study

The theoretical and practical implications of this research are enormous. For the theoretical growth of tourism, this study provides a structured relationship between many components of Malaysian medical tourism and the development of the business. For those working in the hospitality and medical industries, this study can serve as an example for how a positive image of the location, high-quality service, and attentive staff influence customers' perceptions and attitudes toward medical tourism opportunities in Malaysia.

In addition to that, there are practical consequences and suggestions for speciality hospital management based on the findings of this study. People value cleanliness and neatness, professionalism of the employees, their attitude toward patients, and dependability of service delivery more than anything else. Specialized hospitals need to strengthen their non-medical services, including hospitality, sports and leisure, entertainment, and social activities, to acquire a competitive edge in the health tourism industry. They should also match customer expectations based on market trends. That's why specialist hospitals and medical spas need to better grasp the value of extra services they offer to increase their quality. There are several problems with the study that might affect the findings. The research tool is the two most important considerations. Hospital/health services, hotels, and travel/leisure are the three main components of medical tourism. As a result, governments who participate in this developing business might benefit significantly from appealing policies and effective marketing tactics. To do this, host governments must implement a legislative framework that is appealing to consumers throughout the globe. Consumers must be encouraged to go to the host nations to obtain the treatment they seek. Different approaches need to be developed for each because of the uneven participation of the private and public sectors in Malaysia's health tourism industry.

## Limitations and future study recommendation

A small sample size but a representative sample was used for the analysis. The model for evaluating service quality should be evaluated on a more significant sample in the future study, which would enable the application of sophisticated statistical approaches. Patients' expectations and satisfaction could also be analyzed to see statistical significance variations between persons in a healthcare facility due to an injury or recovery and those who utilize medical services to enhance their wellbeing. Further studies should examine the variations in perceived and expected efficiency between non-profit and for-profit medical institutions between private and public-owned facilities and between the perspectives of patients and service providers. New items and questions on the significance of specific characteristics for patients might be added to the study instrument.

## Data availability statement

The raw data supporting the conclusions of this article will be made available by the authors, without undue reservation.

## Author contributions

LL: conceptualizing the idea, introduction, and supervision. NR: literature review. AP: data collection, data analysis, and conclusion. WB: data collection. All authors contributed to the article and approved the submitted version.

## Conflict of interest

The authors declare that the research was conducted in the absence of any commercial or financial relationships that could be construed as a potential conflict of interest.

## Publisher's note

All claims expressed in this article are solely those of the authors and do not necessarily represent those of their affiliated organizations, or those of the publisher, the editors and the reviewers. Any product that may be evaluated in this article, or claim that may be made by its manufacturer, is not guaranteed or endorsed by the publisher.
